# Leader (L) and L* proteins of Theiler's murine encephalomyelitis virus (TMEV) and their regulation of the virus' biological activities

**DOI:** 10.1186/1742-2094-3-19

**Published:** 2006-08-16

**Authors:** Masumi Takano-Maruyama, Yoshiro Ohara, Kunihiko Asakura, Takako Okuwa

**Affiliations:** 1 Department of Microbiology, Kanazawa Medical University, Uchinada, Ishikawa 920-0293, Japan

## Abstract

Theiler's murine encephalomyelitis virus (TMEV) is divided into two subgroups on the basis of their different biological activities. GDVII subgroup strains produce fatal poliomyelitis in mice without virus persistence or demyelination. In contrast, TO subgroup strains induce demyelinating disease with virus persistence in the spinal cords of weanling mice. Two proteins, whose open reading frames are located in the N-terminus of the polyprotein, recently have been reported to be important for TMEV biological activities. One is leader (L) protein and is processed from the most N-terminus of the polyprotein; its function is still unknown. Although the homology of capsid proteins between DA (a representative strain of TO subgroup) and GDVII strains is over 94% at the amino acid level, that of L shows only 85%. Therefore, L is thought to be a key protein for the subgroup-specific biological activities of TMEV. Various studies have demonstrated that L plays important roles in the escape of virus from host immune defenses in the early stage of infection. The second protein is a 17–18 kDa protein, L*, which is synthesized out-of-frame with the polyprotein. Only TO subgroup strains produce L* since GDVII subgroup strains have an ACG rather than AUG at the initiation site and therefore do not synthesize L*. 'Loss and gain of function' experiments demonstrate that L* is essential for virus growth in macrophages, a target cell for TMEV persistence. L* also has been demonstrated to be necessary for TMEV persistence and demyelination. Further analysis of L and L* will help elucidate the pathomechanism(s) of TMEV-induced demyelinating disease.

## Introduction

**Figure 1:**
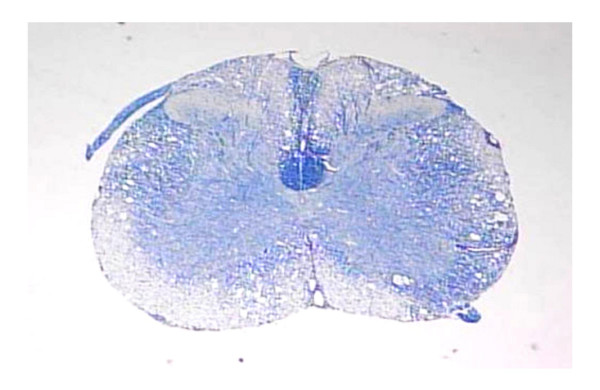
Theiler's murine encephalomyelitis virus (TMEV)-induced demyelination. Spinal cord from a female SJL/J mouse 6 months postinoculation (p.i.) with DA strain of TMEV. Severe demyelination and scattered inflammatory cell infiltration are observed in the white matter (Klüver-Barrera stain, x40).

Theiler's murine encephalomyelitis virus (TMEV) belongs to the genus *Cardiovirus *of the family *Picornaviridae *and is classified into two subgroups of strains [1-4]. Although the sequence identity between strains from these two subgroups is 90.4% at the nucleotide (nt) level and 95.7% at the amino acid (AA) level [5,6], these subgroup strains induce widely different biological activities. GDVII subgroup strains produce acute fatal polioencephalomyelitis in mice without virus persistence or demyelination. On the other hand, TO subgroup strains cause a milder polioencephalomyelitis followed by virus persistence and demyelination. The pathological features of this demyelination are reminiscent of the human demyelinating disease, multiple sclerosis (MS) (Fig. 1) [1-4]. Although several other viruses are known to induce demyelination [7], TMEV-induced demyelinating disease serves as an excellent animal model for MS [1-4]. However, the precise mechanisms of virus persistence and demyelination still remain unknown.

**Figure 2:**
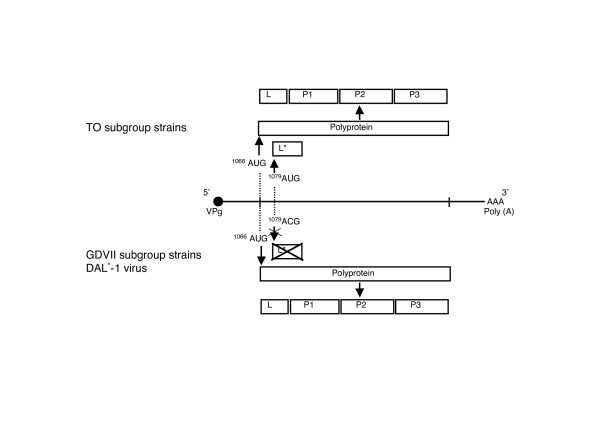
TMEV genome and two different initiation sites. All the TMEVs have an authentic initiation site at nucleotide (nt) 1066, from which the polyprotein is translated followed by cleavage into L, P1, P2 and P3. DA subgroup strains synthesize a small 17–18 kDa protein, L*, from an alternative, out-of-frame, initiation site, which is located at nt 1079. In contrast, GDVII subgroup strains or DAL*-1 virus do not synthesize L* since the L* initiating AUG is replaced with ACG.

Since infectious cDNAs were constructed from the late 1980s to the early 1990s [8-11], various studies using recombinant viruses between GDVII and DA (or BeAn) strains have been carried out to clarify the region responsible for those biological activities. The studies have demonstrated that capsid proteins, especially VP1 and VP2, are important for virus persistence and demyelination [1,3]. In addition to these structural proteins, two proteins designated leader (L) and L* that are located in the N end of the polyprotein (Fig. 2) also play a role in TMEV biological activities [2,3,12].

The present review focuses on the roles of L and L* in regulating the biological activities of TMEV.

### TMEV: properties and biological activities

The TMEV virion is an icosahedron approximately 28 nm in diameter with no lipid-bilayer envelope. A single-stranded RNA is packaged in the shell that consists of four capsid proteins, VP1, VP2, VP3 and VP4 [13]. Neutralizing epitopes have been identified [14-16], the nt and predicted AA sequence determined [5,9,17,18], and full-length infectious cDNAs have been constructed [8-11]. In addition, the three-dimensional structure was resolved by means of X-ray crystallography in the early 1990s [19,20]. The RNA genome is positive sense and approximately 8,100 nt long. An open reading frame (ORF) between the 5' and 3' non-coding regions is translated into a long polyprotein, which is then cleaved into L, P1, P2 and P3. The 5' terminus is covalently linked to VPg, which plays a role in RNA replication. The 3' non-coding region has a poly (A) tract. The coding regions for L and L* are located at the most 5' terminus of the polyprotein coding region (Fig. 2). Details of this will be described later.

GDVII subgroup strains, typified by the GDVII strain, are highly virulent and cause an acute fatal polioencephalomyelitis in mice after intracerebral and peripheral routes of inoculation. After an incubation period of usually less than 2 weeks, infected mice show circling, cachexia, and ruffled hair with a progressive flaccid paralysis. Neither virus persistence nor demyelination is observed in the few surviving mice. Histopathological examination reveals severe necrosis of neurons of the hippocampus, cortex, and spinal cord anterior horn, with microgliosis, neuronophagia, and inflammatory cell infiltration [1-4].

On the other hand, TO subgroup strains cause a biphasic disease after intracerebral inoculation. The early disease, which appears 1–2 weeks postinoculation (p.i.), has clinical and pathological features that are similar to those seen with the GDVII subgroup strains, but milder. Mice recover from the early disease and then develop a chronic, progressive white matter demyelinating disease 1–2 months p.i. Clinical signs include spastic paralysis, inactivity and urinary incontinence. The demyelination mainly affects the spinal cord, with an unexplained sparing of the cerebellar hemispheric white matter. These pathological findings are reminiscent of MS, that is, inflammatory cell infiltration and loss of myelin in the face of relative preservation of axons [1-4]. Therefore, this demyelinating disease is considered to be an excellent animal model for MS, as noted above.

### The target cell for DA persistence

Both subgroup strains of TMEV infect mainly neurons during the acute stage of infection [1-4]. It is of interest that DA viral antigen and RNA that are present in neurons during the acute stage of infection disappear from neurons in the chronic demyelinating stage, presumably because these cells are cleared, perhaps by apoptosis. The cellular localization of DA viral antigen and RNA in the chronic demyelinating stage is somewhat controversial. There are two proposals: viral persistence in oligodendrocytes and/or viral persistence in macrophages. Immunoelectron microscopic study have shown viral antigen in oligodendrocytes at 45 days p.i. or later. Based on this, Rodriguez and coworkers proposed that a "gdying-back" process might occur in virus-infected oligodendrocytes [21,22], resulting in demyelination. In nude mice, demyelination occurs without evidence of myelin stripping by macrophages, suggesting that the demyelination occurs secondary to a lytic infection of oligodendrocytes [23]. Also, in nude mice, electron microscopic studies have demonstrated paracrystalline arrays of picornavirus in degenerating glial cells, many of which were identified as oligodendrocytes. A lytic infection of oligodendrocytes has been proposed as a cause of the demyelination [24]. On the other hand, a number of studies have found that the virus persists in macrophages. Using ultrastructural immnohistochemical techniques, researchers have observed viral inclusions in macrophages in and around demyelinating lesions [25]. Two-color immunofluorescent staining has shown that viral antigen is predominantly within macrophages infiltrating demyelinating lesions [26]. Infectious virus can be recovered from infiltrating mononuclear cells isolated directly from the central nervous system (CNS) [27]. Cultured primary brain macrophages can be efficiently infected with the DA strain without the induction of a significant cytopathic effect [28]. The importance of macrophages in late demyelinating disease is further emphasized by the observation that depletion of blood-borne macrophages by dichloromethylene diphosphonate prevents virus persistence in mice infected with the DA strain [29]. From these data, it appears likely that macrophages are the major cells containing persistent viral genome. Therefore, the mechanism by which DA survives in macrophages may clarify DA persistence.

### L and picornaviruses

The aphthoviruses and caridioviruses are the only members of the *Picornaviridae *family that contains an L coding region at the most 5' terminus of the ORF [13]. In the case of foot-and-mouth disease virus (FMDV), an aphthovirus, L has two proteolytic functions. One is autocatalytic cleavage from the viral polyprotein [30,31], and the second is cleavage of the p220 component of the cap-binding protein complex [32], resulting in the shut off of host protein synthesis. On the other hand, the function of L of the cardioviruses is not well defined.

L has been implicated in other functions of picornaviruses. FMDV L is involved in inhibiting phosphorylation of eukaryotic initiation factor 2 by double-strand RNA-dependent protein kinase [33]. L of cardioviruses inhibits the expression of alpha/beta interferon (IFN *α*/*β*) (see later discussion), which is a critical tool to inhibit viral spread. L of mengovirus, which also belongs to the genus *Cardiovirus*, has been reported to inhibit the iron/ferritin-mediated activation of NFκB [34]. The functions of L of TMEV and the other cardioviruses remain incompletely clarified.

### The properties of TMEV L

TMEV L, which is processed from the most N-terminus of the polyprotein, consists of 76 amino acids [17,18] (Fig. 2). The release of L occurs rapidly. However, it lacks autocatalytic activity [35].

Although the identity of capsid proteins between DA and GDVII strains is over 94% at the AA level, that of L shows only 85% identity [5,6]; the low identity of the AA sequence of L between both TMEV subgroups suggests that L may contribute to the determination of the DA subgroup-specific biological activities, such as attenuated neurovirulence, viral persistence and demyelination. In addition, the identity of TMEV L with L of EMCV, which belongs to the same cardiovirus genus, is ~35 % although that of the entire coding region is about 60%. TMEV L contains a zinc-binding motif Cys-His-Cys-Cys that, interestingly, is present in L from all the cardioviruses [6,36].

Our unpublished data demonstrate that L is synthesized with the same kinetics as capsid proteins and is not incorporated into the virion. L is synthesized in the cytoplasm of host cells and, in part, transferred into the nucleus [37]. The data suggest that L may function through its interactions with cellular protein(s).

### The biological activities of TMEV L

A mutant DA or GDVII virus with a deletion of L causes poor growth in the L929 mouse fibroblast cell line that produces IFN *α*/*β*, but not in BHK-21 cells producing no IFN *α*/*β *[38,39]. Since L of DA strain has been reported to inhibit the transcription of IFN *α*/*β *[36,40], the replication of virus is suppressed under pressure of IFN *α*/*β*. L prevents transcription of IFN *α*/*β *because of its interference with the nuclear localization of IFN regulatory factor 3 (IRF-3), a transcription factor required for the expression of IFN *α*/*β *[37]. The zinc-binding motif within L directly binds zinc ions and is a key factor in the inhibition of IFN *α*/*β *expression [36,37,40,41].

The importance of IFN *α*/*β *in the animal's host cell defense from TMEV infection has been demonstrated in TMEV infections of IFN *α*/*β *receptor-deficient mice [42]. These mice die of overwhelming encephalomyelitis following intracerebral inoculation with DA strain because of enhanced virus replication. Similarly, DA virus with a mutation in the zinc-binding motif of L is cleared from the CNS since the mutation induces the transcription of IFN *α*/*β*, resulting in production of IFN *α*/*β *[36].

The inhibition of IFN *α*/*β *by L, however, is not enough to allow TMEV to escape all host defense mechanisms. Indeed, DA strain is cleared after the first phase of disease in genetically resistant C57BL/6 mice in which L is expressed. Disruption of *β*_2_-microglobulin gene in resistant mice, which fail to express class I MHC molecules and therefore lack CD8^+ ^T lymphocytes, abrogates resistance to the DA strain, allowing the virus to persist [43]. This report suggests that class I-restricted CD8^+ ^T lymphocytes are important for persistent infection, in addition to inhibition of IFN *α*/*β *by L.

### The properties of L*

During an investigation of polyprotein processing, Roos et al identified a small 17–18 kDa protein that is synthesized *in vitro *in rabbit reticulocyte lysates programmed from *in vitro*-derived transcripts of full-length clones of DA strain cDNA [35]. DA subgroup strains have an alternative translation initiation site at nt 1079, in addition to the authentic initiation site for the polyprotein at nt 1066 [44] (Fig. 2). This alternative initiation site is out-of-frame with the polyprotein and is used to translate the 17–18 kDa protein, designated L*. The synthesis of L* is TO subgroup-specific because this alternative initiation site is not present in GDVII subgroup strains (where the L* AUG is substituted by an ACG) (Fig. 2) [6,44]. Therefore, this DA subgroup-specific out-of-frame protein is thought to play an important role in characterizing the different biological activities of TMEV subgroups, especially viral persistence and demyelination.

There were initial difficulties in generating an anti-L* antibody, perhaps related to a relative lack of antigenicity or to extreme hydrophobicity resulting in solubility problems. These difficulties were overcome with the production of a rabbit polyclonal antibody against synthetic peptides corresponding to amino acid residues 70–88 (the computer-predicted antigenic site) [45,46]. Studies employing this antibody have demonstrated that L* is synthesized with kinetics similar to that of other viral proteins, although in a lesser amount. After synthesis, L* remains stable in the cytoplasm and is not incorporated into virions. Immunofluorescent staining and immunoblotting of microtubule preparations have demonstrated that L* is associated with microtubules. Experiments employing transient expression of L* have suggested that the 5' one third of the L* coding region is responsible for this association [46].

### The role of L* in virus growth in macrophages

We examined the growth patterns of DA (which persists in the CNS) and GDVII (which does not persist) strains in J774-1 cells, a representative mouse macrophage cell line, since macrophages are the target cells for virus persistence, as described above [25-29]. The growth curves clearly demonstrated that DA strain grows well in J774-1 cells, while GDVII strain does not. On the other hand, both strains grew well equally in BHK-21 cells [47]. These results are of interest since virus growth is necessary for the maintenance of the viral genome, which is essential for virus persistence [48]. TMEV subgroup-specific virus growth was studied in various other cell lines including neural cells. The results demonstrated that enhanced DA growth compared to GDVII is only observed in macrophage cell lines. Therefore, the TMEV subgroup-specific virus growth is also host cell-dependent [49].

The role of L* in TMEV subgroup-specific virus growth was further studied in a 'loss of function' experiment using a mutant virus, DAL*-1, which has an ACG rather than AUG at the initiation site of L* coding region, and therefore does not synthesize L*. Takata et al found that the DAL*-1 virus failed to grow in J774-1 cells, whereas the virus grew well in BHK-21 cells [50]. In addition, DAL*-1 virus failed to grow in other macrophage cell lines, suggesting that L* plays an important role in host cell-dependent subgroup-specific infection [49].

In order to carry out a 'gain of function' experiment to further confirm the role of L* in host cell-dependent subgroup-specific virus growth, Obuchi et al constructed a recombinant virus, DANCL*/GD, which has DA 5' noncoding and L* coding regions in the background of GDVII (with synthesis of L*). DANCL*/GD virus had enhanced growth activity in J774-1 cells compared to GDVII, suggesting that L* is important for the subgroup-specific virus growth in macrophages [45]. However, a pitfall of L* in studies such as these involving L* is that the sequence of L* overlaps with that of polyprotein (L and a part of P1). Therefore, it is impossible to evaluate the role of L* without affecting the nt sequence of the corresponding coding region of the polyprotein. In order to overcome this situation, we recently established an L*-expressing J774-1 cell line. GDVII and DAL*-1 viruses do not grow in control cells, which do not express L*, whereas virus growth is enhanced in L*-expressing cells [51]. van Eyll et al also have shown that L* ORF is required for virus growth in macrophage cell lines [52]. Therefore, L* is essential for host cell-dependent subgroup-specific virus growth, which is likely to play an important role in TMEV pathogenesis.

### L* and apoptosis of macrophages

TMEV is reported to induce apoptosis *in vitro *and *in vivo*. Tsunoda et al detected the apoptosis *in vivo *and suggested that the apoptosis of neurons may be responsible for the fatal outcome of GDVII infection [53]. Apoptosis has also been found during the chronic stage of DA infection [54]. Of interest, the majority of apoptotic cells (CD3^+ ^T cells) were uninfected, suggesting an activation-induced cell death.

The role of L* in apoptosis is studied in both 'loss of function' and 'gain of function' experiments. In a 'loss of function' experiment, a macrophage cell line, P388D1, was infected by wild type DA (which synthesizes L*) as well as DAL*-1 and GDVII viruses (neither of which synthesizes L*). DAL*-1 and GDVII viruses induced DNA laddering 12 hrs p.i., however, wild type DA did not. TUNEL-staining demonstrated that DAL*-1 and GDVII viruses caused apoptosis in 38% and 43% of P388D1 cells, respectively, while only 6% of DA-infected cells were apoptotic. These studies suggest that L* has an anti-apoptotic activity in macrophage cells [55]. In contrast to these findings, TMEV infection of microglia does not induce apoptosis [56]. The differing results may relate to special properties of microglia that are distinct from those of circulating macrophages.

Himeda et al established L*-expressing P388D1 cells to confirm the anti-apoptotic activity of L* in a 'gain of function' experiment [57]. DAL*-1 virus induced prominent DNA laddering in control cells that do not express L*, but failed to do so in L*-expressing P388D1 cells. The activity of caspase-3 was raised in the control cells and was inhibited by a caspase family inhibitor, Z-VAD-FMK, whereas caspase activity was significantly decreased in L*-expressing cells. The authors speculate that the major apoptotic pathway following TMEV infection may be a death receptor-mediated pathway since no cytochrome c release was detected.

### The role of L* in virus persistence and demyelination

A challenging issue that remains is whether L* plays a role in virus persistence and demyelination. An initial question that required answering is whether L* is expressed *in vivo*. Asakura et al first demonstrated the expression of L* *in vivo *by means of immunoprecipitation and immunoblotting studies using anti-L* antibody [58]. These studies also localized L* in the mouse CNS during the acute stage of infection. L* was identified in neurons and colocalized with capsid protein, VP1.

L* was found to play an important role in virus persistence and demyelination by employing a 'loss of function' experiment. DAL*-1 virus produces an early acute polioencephalomyelitis similar to the parental DA, however, the viral RNA genome is no longer detected in the spinal cord of mice 6 weeks p.i. [55]. In addition, there is minimal if any evidence of demyelination or inflammation in the spinal cord [59]. L* appears to inhibit the generation of H-2K-restricted TMEV-specific cytotoxic T cells, therefore permitting a persistent infection to occur in susceptible mouse strains [60]. However, it is also reported that wild type-DA (which expresses L*) induces H-2K-restricted TMEV-specific cytotoxic T cells [61], In addition, the above findings regarding L* were also called into question by Michiels and colleagues because the absence of the L* AUG initiation codon in a mutant DAL*-1 virus generated from a different DA infectious clone had only a weak influence on virus persistence [62]. The discrepancy is due to one nt sequence of the two viruses (Roos, R., personal communication). Further studies by van Eyll et al [52] using DA virus mutants with a stop codon in the L* reading frame (leading to a truncated L*) confirmed the key role of L* in virus persistence and demyelination.

Yamasaki et al. reported a utilization of the L* translation initiation vs. the polyprotein's AUG [63]. These investigators proposed that L* (rather than the polyprotein) is preferentially synthesized in certain CNS cells (e.g. microglial cells) following infection with DA subgroup strains. The production of only small amounts of capsid protein in certain cells would foster virus persistence and lead to restricted expression of the virus in the chronic stage.

The above data indicate that L* is a key determinant of TMEV persistence, subsequently leading to an inflammatory demyelination in the CNS, similar to that in MS. However, all the *in vivo *data that have been obtained to date are from 'loss of function' experiments. Additional data through by 'gain of function' experiments, such as those involving L*-expressing transgenic mice, would be valuable in order to confirm the role of L* protein *in vivo*.

From the above data regarding L and L*, it is speculated that DA strain could escape from host immune defense(s) through the inhibition of IFN *α*/*β *by L in the early stage of infection. DA that had escaped from early immune attack could then maintain its genome in macrophages with the aid of L* in the chronic stage of infection. The presence of TMEV genome in macrophages could trigger a cascade of immune system, leading to immune-mediated demyelination.

## Conclusion

Both DA and GDVII subgroup strains of TMEV synthesize L, which consists of 76 AA and is processed from the most N-terminus of the polyprotein. L contains a zinc-binding motif, Cys-His-Cys-Cys, which is conserved among all cardioviruses and directly binds to zinc ions. L prevents transcription of IFN *α*/*β *through interference of the nuclear localization of IRF-3, a transcription factor important for the expression of IFN *α*/*β*.

DA subgroup strains synthesize L*, which is out of frame with the polyprotein. GDVII subgroup strains have an ACG rather than AUG corresponding to the initiation codon of L*, resulting in no synthesis of L*. A 'loss of function' experiment using mutant DA virus that fails to synthesize L*, as well as a 'gain of function' experiment using an L*-expressing macrophage cell line, demonstrated that L* has anti-apoptotic activity and is required for virus growth in macrophages. *In vivo *experiments using mutant DA virus, in which L or L* is not synthesized, also demonstrated that these are key proteins regulating the DA subgroup-specific biological activities, i.e., virus persistence and demyelination. Further studies clarifying the roles of L and L* will elucidate the pathomechanism(s) of TMEV-induced demyelinating disease, and may also provide insights into our understanding for MS.

## Abbreviations

L: leader protein

L*: L* protein

TMEV: Theiler's murine encephalomyelitis virus

CNS: central nervous system

## Competing interests

The author(s) declare that they have no competing interests.

## Authors' contributions

MT conceived this review and wrote the initial draft with KA under the direction of YO. YO and TO modified, wrote and submitted the final draft. All authors read and approved the final version.
